# The effect of adipose tissue-derived stem cells in a middle cerebral artery occlusion stroke model depends on their engraftment rate

**DOI:** 10.1186/s13287-017-0545-y

**Published:** 2017-04-26

**Authors:** Saskia Grudzenski, Sebastian Baier, Anne Ebert, Pim Pullens, Andreas Lemke, Karen Bieback, Rick M. Dijkhuizen, Lothar R. Schad, Angelika Alonso, Michael G. Hennerici, Marc Fatar

**Affiliations:** 1 Department of Neurology, Universitätsmedizin Mannheim, Heidelberg University, Theodor-Kutzer-Ufer 1-3, D-68167 Mannheim, Germany; 2 Computer Assisted Clinical Medicine, Medical Faculty Mannheim, Heidelberg University, 68167 Mannheim, Germany; 3 Department of Radiology, UZ-Brussel, Vrije Universiteit (VUB), 1090 Brussels, Belgium; 4 Institute of Transfusion Medicine and Immunology, Medical Faculty Mannheim, Heidelberg University, 68167 Mannheim, Germany; 5 Biomedical MR Imaging and Spectroscopy Group, Image Sciences Institute, University Medical Center Utrecht, 3584 CX Utrecht, The Netherlands

**Keywords:** Adipose tissue-derived stem cell, Cell tracking, Cell engraftment, Focal ischaemia, High-field MRI, MCA occlusion, Rodent model

## Abstract

**Background:**

In the field of experimental stem cell therapy, intra-arterial (IA) delivery yields the best results concerning, for example, migrated cell number at the targeted site. However, IA application also appears to be associated with increased mortality rates and infarction. Since many rodent studies systemically apply 1 × 10^6^ cells, this could also be a consequence of engrafted cell number. The aim of this study was therefore to investigate the effect of different doses of adipose tissue-derived stem cells (ASCs) on engraftment rates and stroke outcome measured in vivo using 9.4-T high-field magnetic resonance imaging (MRI).

**Methods:**

Male Wistar rats (*n* = 43) underwent a middle cerebral artery occlusion (MCAo) for 45 or 90 min, followed by IA delivery of either saline or 1 × 10^6^, 3 × 10^5^, or 5 × 10^4^ ASCs pre-labelled with very small superparamagnetic iron oxide particles (VSOPs). MRI (9.4-T) analysis was performed 48 h and 9 days post-MCAo. Lesion volumes were assessed by analysis of T2-weighted images and cell signal tracking showing cell engraftment and active cell migration by an improved T2*-analysis.

**Results:**

The ASC-derived signal intensity increased in the affected hemisphere 48 h post MCAo with injected cell number (*p* < 0.05). The analysis of stroke volumes revealed an increased infarction after injection of 1 × 10^6^ ASCs compared to controls or application of 5 × 10^4^ ASCs (*p* < 0.05). At 9 days post-MCAo, injection of 3 × 10^5^ ASCs resulted in reduced infarct volumes (*p* < 0.05). Correspondingly, MRI analysis revealed no changes in cell numbers between both MRI examinations but showed active ASC migration to the site of infarction.

**Conclusion:**

Our results confirm that IA injection is an efficient way of targeting damaged brain tissue but its usefulness strongly depends on the right dose of delivered stem cells since this factor has a strong influence on migration rate and infarct volume, with better results for doses below 1 × 10^6^ cells. Future challenges will include the determination of therapeutic doses for best cellular engraftment and stroke outcome.

**Electronic supplementary material:**

The online version of this article (doi:10.1186/s13287-017-0545-y) contains supplementary material, which is available to authorized users.

## Background

Stem cell (SC) therapy has gained tremendous attention as the expectation in regenerative medicine seems to be obvious: to replace diseased or dying tissue areas with new healthy cells. Adipose tissue-derived stem cells (ASCs) can be extensively extracted from subcutaneous human adult adipose tissue and are increasingly being recognised for their proliferating capacity in cell culture as well as for their multipotency [1–4]. Additionally, their immune-privileged and immunomodulatory properties potentially allow for allogeneic transplantation into immunocompetent recipients and support tissue repair through immunosuppressive effects [5–10]. Furthermore, ASCs secrete numerous trophic factors which modulate inflammation, remodelling, and apoptosis, e.g., during stroke. This suggests they can be used as an alternative to largely unsuccessful drug approaches [9, 11]. However, a lot of important questions in this promising field of non-drug therapy have still not been answered sufficiently; for example, what is the best delivery route or the right dose of cells for application. Regarding the route of application, results indicate that, in contrast to locally transplanted SCs, systemically (that is, intravenously (IV) or intra-arterially (IA)) injected cells have potent anti-inflammatory effects, promote brain remodelling, and induce functional neurological recovery [12]. However, there are also differences within systemic application routes that can dramatically affect cell engraftment. IV injection is a minimally invasive and therefore clinically highly relevant technique, but one very important point in the field of SC therapy is the question of how to bring enough active components, that is SCs, to the target tissue. With regards to engraftment rates, IV injection leads to delayed and poor engraftment due to long-distance migration, and filtering organs (predominantly the lungs) trapping a large amount of cells [13, 14]. More promising results concerning the migration, distribution, and cell number at the target site have been obtained after IA injection. However, it seems that this benefit comes at the cost of higher mortality rates and even increased infarction [14–16]. It is questionable if this observation is necessarily linked to IA administration or to the amount of SCs injected since, despite the fact that experimental details have gained more attention in recent studies, individual settings still differ significantly and have not been tested sufficiently, especially the correct amount, that is the therapeutic dose of cells. Most of the recent studies engrafting SCs in rodents systemically used 1 × 10^6^ cells [14, 16–18]. It will therefore be important to question how much influence this parameter has on SC therapy, especially on mortality rate, traceability of engrafted cells, and infarct size. Until now, no study has ever investigated the relationship between different doses of IA delivered stem cells, cell engraftment, and migration behaviour, as well as the effect on stroke outcome, although this is of tremendous importance with regard to stem cell treatment in humans. In this study, we have investigated the effect of different amounts of ASCs, pre-labelled with very small superparamagnetic iron oxide particles (VSOP), on cell migration and distribution as well as the effect on infarct size in vivo in rat brain parenchyma using 9.4-T small animal magnetic resonance imaging (MRI) at two given times. Cells were injected IA into the external carotid artery after middle cerebral artery occlusion (MCAo).

## Methods

### Cell culture and ASC labelling

Cells were processed and characterized as described previously and cultured in 5% CO_2_ at 37 °C in mesenchymal stem cell (MSC) growth medium supplemented with 10% MSC growth supplement, 2% l-glutamine, and 0.1% gentamicin sulphate/amphotericin-B (MSCGM Single Quots, Lonza, Walkersville, MD, USA) [19]. Mycoplasma contaminations were excluded by regular tests during cell culture. Subcultivation was performed at densities of 2 × 10^5^ cells per 75 cm^2^ cell culture flask to prevent confluency. For experiments, the passage number was below ten. Before engraftment, ASCs were labelled with electrostatically stabilized VSOPs (VSOP 5 nm Flu vitro, Ferropharm, Teltow, Germany) as described previously. VSOPs have been proven to be better suited for in vivo cell labelling due to their monomer citrate coating resulting in diameters of only 4–9 nm and the easy and stable labelling of cells via endocytosis [20, 21]. To verify optimal growth behaviour, and to exclude any cytotoxic effect caused by cell labelling, growth curves were obtained by seeding 5 × 10^5^ VSOP labelled and unlabelled ASCs and assessing vital and dead cells up to 14 days. The test was performed five times. Additionally, possible VSOP-mediated cytotoxicity was determined by measuring the release of lactate dehydrogenase (LDH) using the Cytotoxicity Detection Kit (Roche, Mannheim, Germany). Unlabelled ASCs served as negative controls and ASCs treated with 2% Triton X as positive controls. Values were calculated by subtracting the background control (cell culture medium) and mean values from three seeded wells for each point (negative control = unlabelled cells; positive control = unlabelled cells re-suspended with cytotoxic 2% Triton and cells labelled with 3 mM VSOPs) were calculated. The test was performed four times. Results are described in Additional file 1: S-1 and shown in Additional file 2: Figure S1A and B.

### Animals

This manuscript was written in accordance with the ARRIVE (Animal research: reporting in vivo experiments) guidelines [22]. Male Wistar rats originated from Charles River Laboratories (Sulzfeld, Germany) and were kept in the Central Facility for Medical Research (ZMF) of the University Medical Center, Mannheim, under a 12 h light cycle with food and drinking water available ad libitum. Animals were immunocompetent and did not receive immunosuppressive therapy.

### MCAo via filament model and cell application via the external carotid artery

Anaesthesia was introduced with isoflurane 2.5% vaporised in O_2_ (med oxygen) in 43 male Wistar rats at the age of 12 weeks and with a weight of 383 ± 76.2 g. Anaesthesia was maintained at 1.5% during surgery (Abbott, Wiesbaden, Germany) via a face mask. Body temperature was maintained at 37 °C with an electric heating pad and feedback control via a rectal temperature probe. Arterial blood gases (pO_2_, pCO_2_, pH, oxygen saturation) and heart rate were controlled using the radiometer ABL80 Flex (Radiometer GmbH, Willich, Germany).

Right-sided MCAo via the external carotid artery (ECA) was performed as described previsouly using silicon rubber-coated monofilaments with a diameter of 390 μm (Doccol Corporation, Sharon, MA, USA) [23, 24]. Non-invasive cerebral blood flow (CBF) measurements to ensure vessel occlusion were performed by laser Doppler measurement of the affected right hemisphere using the moor VMS-PC V2.0 system (Moor Instruments, Millway, UK). Only animals with visible vessel occlusion and a significant drop in CBF were included in the study.

Due to the high mortality rates after MCAo of 90 min and administration of 1 × 10^6^ cells, we included animals with a reduced occlusion time of 45 min for all groups. The group of animals receiving 5 × 10^4^ cells all underwent MCAo for 45 min. After MCAo, 0.5 ml NaCl 0.9% was injected containing no cells (control animals; MCAo 45 min, *n* = 8; MCAo 90 min, *n* = 9), 1 × 10^6^ ASCs (MCAo 45 min, *n* = 4; MCAo 90 min, *n* = 3), 3 × 10^5^ ASCs (MCAo 45 min, *n* = 6; MCAo 90 min, *n* = 6), or 5 × 10^4^ ASCs (*n* = 7; all MCAo 45 min). After rinsing with 0.15 ml NaCl 0.9%, the ECA was ligated permanently and ligation on the common carotid artery was removed to allow reperfusion. The laser Doppler probe was removed and both wounds were closed. Mortality rates are described in Additional file 1: S-2.

### MRI measurements

Infarct localization and cell signals were measured 48 h and 9 days post MCAo in vivo using a 9.4-T Biospec 94/20 USR small animal system equipped with 740 mT/m gradients and a 1H surface cryogenic probe (Bruker, Ettlingen, Germany). An ischaemic lesion was defined based on hyperintense T2-weighted images.

Diffusion-weighted images (DWIs) and the corresponding maps of apparent diffusion coefficient (ADC) were obtained to identify signals obtained by T2- and T2*-weighted images as an infarct and tissue developments such as necrosis and oedema. DWIs were acquired using a respiratory-triggered single-shot spinecho echo-planar imaging (SS-EPI) sequence using the following parameters: 30 gradient directions and seven b-values (b = 0, 500, 1000, 1500, 2000, 2500, 3000 s/mm^2^) along each direction, Δ/δ = 10/2.5 ms, TR/TE = 3000/21.5 ms, FOV = 14 × 11 mm, matrix size = 100 × 78, 20 slices with a slice thickness/spacing of 0.8/0.2 mm, bandwidth = 300000 Hz, NEX = 1, and a total acquisition time of 9 min. ADC maps were calculated using Bruker software. T2-weighted images were acquired with a coronal T2-weighted RARE sequence using the same geometry as the SS-EPI: TR/TE = 3300/60 ms, echo train length = 4, FOV = 14 × 11 mm, matrix size = 320 × 256, bandwidth = 110000 Hz, NEX = 4, and a total acquisition time of 4 min.

Labelling cells with VSOPs leads to a strong decrease in the transverse relaxation time of water protons diffusing close to the cells, resulting in turn in signal loss in T2*-weighted gradient echo images [25]. Thus VSOP-labelled cells can be visualized by decreased signals in T2*-weighted gradient echo images. Visualization was realised by an axial gradient-echo FLASH sequence (TR = 50 ms, TE = 8.7 ms, FA = 15_, FOV = 26.88 × 17.92 × 8.96 mm^3^, MA = 768 × 512 × 256, NA = 2, t_acq_ = 2 h 23 min, Res = 35 × 35 × 35 μm^3^).

### MRI analysis of infarct sizes and cell signal intensity

All analyses were performed blind to the treatment protocol or functional outcome of the tested animals. MRI analysis was performed 48 h post-stroke. The difference in cell signal intensity (ΔCSI) was measured 48 h post MCAo in seven T2*-weighted MR images per animal, exactly defined by anatomical structures. The affected and contralateral hemisphere was defined as the region of interest (ROI) and mean signal intensity as well as standard deviation (SD) were measured within each ROI using Image J software (National Institute of Health, MD, USA). The mean ΔCSI between affected (affect. hemi) and contralateral hemisphere (contralat. hemi) was then calculated and averaged over seven slices using the following formula:

$$ \varDelta CSI=\sqrt{}{\left(\frac{SD- ROIcontralat. hemi}{mean- ROIcontralat. hemi}\right)}^2+{\left(\frac{SD- ROIaffect. hemi}{mean- ROIaffect. hemi}\right)}^2 $$

For infarct analysis, twenty T2-weighted MR images per individual were obtained and analysed. The hyperintense infarct area of every MRI slide was calculated using Image J. The area was then multiplied by slice thickness to calculate infarct volumes per MRI slide. The infarct area of the interslice gaps was calculated as the mean infarct area of two neighbour slices. The volumes of interslice gaps were then also calculated by multiplying the intergap thickness by the mean infarct area of two neighbour slices. Final infarct volumes were obtained by adding all the volumes up and were calculated as a percentage of the total individual brain volume of each animal (which was calculated in the same way).

Residual infarct sizes 9 days post-MCAo were calculated as the percentage of infarct sizes obtained after 48 h to determine individual changes in infarct size in each animal. Due to signal changes within 9 days caused by tissue transformation, ambiguous hypointense signals in T2*-weighted images had to be re-analysed by deleting unspecific signals and leaving ASC-derived hypointense voxels. Therefore, all obtained T2*-weighted images per animal (twenty MRI slices corresponding to the numbers of T2-weighted images) were corrected for inhomogeneities using bias field correction [26]. Brains were segmented using a customized version of the brain extraction tool in FSL (Oxford Centre for Functional Magnetic Resonance Imaging of the Brain (FMRIB)’s Software Library). Images were normalized by setting the individual image mean to 1000. All brains were aligned using FSL-FLIRT (FMRIB’s Linear Image Registration Tool) as described previously [27]. In Matlab (The MathWorks Inc., Natick, MA, USA) the de-noised (median filter) image was subtracted from the original image, and the absolute value of the result was taken. Finally, a wiener filter (local noise reduction) was applied. Voxels in the affected hemisphere having intensities higher than the mean + 2SD of voxel intensity in the right hemisphere were considered speckled voxels. When overlaid on the original data, these voxels contained both hyper- and hypointense voxels. Based on a histogram analysis, only the hypointense voxels were selected and considered as ASC-derived signals.

### Histology

After incubation of harvested brains under deep isoflurane anaesthesia by transcardial perfusion with 4% acid-free formaldehyde overnight, 2-mm blocks were dehydrated in ethanol and xylol (Roth, Karlsruhe, Germany) and embedded in paraffin. For tissue staining procedures, 4-μm sections were dewaxed in xylene and rehydrated in alcohol and distilled water. Prussian blue (PB) staining was performed using the Accustain® Iron Stain Kit according to the manufacturer’s guidelines (Sigma-Aldrich, St. Louis, MO, USA). Nuclei were counterstained using nuclear fast red 0.1% (Merck, Darmstadt, Germany) for 10 min. For 3,3′-diaminobenzidine (DAB) and fluorescent staining, antigen retrieval was performed using either citrate buffer (DAKO REAL target retrieval solution, pH 6.0; DAKO, Hamburg, Germany) or tris (hydroxymethyl)aminomethane/ethylenediaminetetraacetic acid (TRIS/EDTA) buffer, pH 9.0 (TRIS, Merck, Darmstadt, Germany; EDTA, SERVA electrophoresis, Heidelberg, Germany) depending on the antibody used. For displaying VSOP fluorescence after rehydration in alcohol, sections were incubated with 4′,6-diamidino-2-phenylindole (DAPI; 1:500; KPL, Gaithersburg, USA) overnight at 4 °C, washed thoroughly with 1× phosphate-buffered saline (PBS), pH 7.4, and mounted in Mowiol (Roth, Karlsruhe, Germany). Results are described and representative images of ASC identification and localisation via staining of PB and VSOP fluorescence are shown in Additional file 1: S-3 and Additional file 3: Figure S2.

DAB staining was performed according to the manufacturer’s guidelines using the ImmPACT DAB Kit (Vector Laboratories, Burlingame, CA, USA). Antibody dilutions were 1:100 for anti-Ki67 (#MAB4190) and 1:30 for anti-human mitochondria (#MAB1273) (both monoclonal, mouse; Merck Millipore, Darmstadt, Germany). Cells were counterstained with hematoxylin (Hollborn&Soehne, Leipzig, Germany). Results and representative images of human cell identification as well as viability testing via staining of human mitochondria and Ki67 are shown in Additional file 1: S-3 and Additional file 4: Figure S3A and B. After dehydrating steps in alcohol, PB and DAB stained sections were preserved in Eukitt (O. Kindler GmbH & CO, Freiburg, Germany).

### Microscopic imaging, datasets, and statistics

A Leica DM 4500 B fluorescence microscope and Leica IM50 Image Manager Software were used (Leica, Wetzlar, Germany) for bright field and image acquisition.

The dataset supporting the conclusions of this article are included within the article (see data ranges, medians, and interquartile ranges (IQRs) in the Tables). For statistical analysis, a standard software package (SPSS for windows, “SPPS Inc.”, Chicago, IL, USA) was used and the two-sided Mann-Whitney *U* test was chosen for calculation of statistical comparisons. A *p* value ≤0.05 was considered significant.

## Results

### MRI analysis of ASC-derived signals 48 h post MCAo

**Fig. 1:**
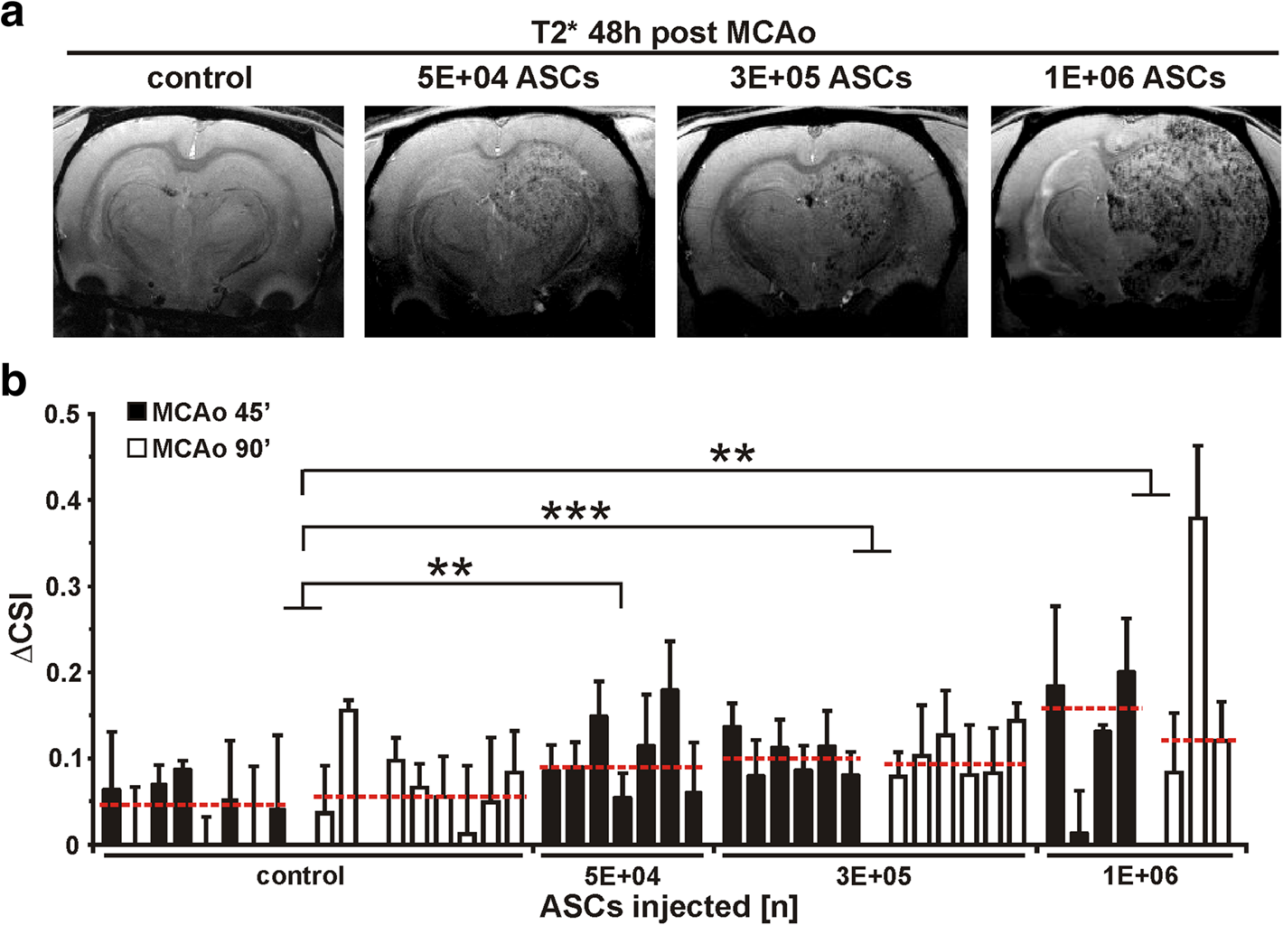
MRI analysis of ASC-derived signals 48 h post-MCAo. **a** Representative T2*-weighted images show hypointense ASC-derived signals in animals representing the control group as well as the group treated with 5 × 10^4^, 3 × 10^5^, or 1 × 10^6^ ASCs. **b** Differences in ΔCSI obtained 48 h post MCAo are shown for all treatment groups. Groups are controls (*n* = 8 for MCAo = 45 min; *n* = 9 for MCAo = 90 min), and animals treated with 5 × 10^4^ ASCs (*n* = 7; all MCAo = 45 min), 3 × 10^5^ ASCs (both *n* = 6 for MCAo 45 min and 90 min), and 1 × 10^6^ ASCs (*n* = 4 for MCAo = 45 min; *n* = 3 for MCAo = 90 min). The median of each treatment group is indicated as a *red dotted line*. Each column represents one animal with mean ΔCSI obtained in seven brain slices per animal ± standard deviation. **p < 0.01 for control group vs. 1 × 10^6^ and 5 × 10^4^ ASC group, ***p < 0.001 for control group vs. 3 × 10^5^ ASC group. *ASC* adipose tissue derived stem cell, *ΔCSI* difference in cell signal intensity, *MCAo* middle cerebral artery occlusion

Forty-eight hours after IA transplantation, ASCs were distributed discretely throughout the entire lesion area in the ipsilateral side of the brain as shown by hypointense dots in representative T2*-weighted images. The intensity of the hypointense signal increased visibly with the number of cells injected (Fig. 1a). To quantify this effect, the mean ΔCSI was calculated for each animal to evaluate differences in CSI between the ischaemic and non-ischaemic hemisphere (for data range, medians, and IQRs for ΔCSI, see Table 1). Results showed that MCAo duration did not affect ΔCSI for the control group and the group receiving 3 × 10^5^ cells. For animals receiving 1 × 10^6^ cells, the occlusion times could not be compared due to restricted data for the group undergoing MCAo for 90 min; however, due to a comparable ΔCSI in the first-mentioned groups, a statistical comparison was performed for every group independently of occlusion time. ΔCSI values were significantly lower for control animals than for animals treated with 5 × 10^4^ cells (*p* < 0.01), 3 × 10^5^ cells (*p* < 0.001), or 1 × 10^6^ cells (*p* < 0.01) (Fig. 1b).

**Table 1:** Group sizes, data range, median, and IQR for ΔCSI and infarct sizes obtained 48 h post MCAo

Variable			ΔCSI			Infarct size (%)		
Treatment (number of ASCs)	MCAo (min)	Group size (*n*)	Data range	Median	IQR	Data range	Median	IQR
Control	45	8	−0.032 to 0.087	0.047	0.078	0.7 to 15.8	6.96	9.25
	90	9	−0.143 to 0.156	0.055	0.066	1.1 to 17.2	3.23	6.76
5 × 10^4^	45	7	0.054 to 0.179	0.089	0.088	0.5 to 9.3	4.63	5.94
3 × 10^5^	45	6	0.079 to 0.136	0.099	0.034	1.7 to 10	5.86	3.83
	90	6	0.079 to 0.143	0.093	0.046	1.1 to 13.1	6.82	6.43
1 × 10^6^	45	4	0.014 to 0.2	0.157	0.12	3.8 to 26.1	13.37	16.93
	90	3	0.083 to 0.379	0.12	–	4.2 to 20.6	19.64	–

*ASC* adipose tissue-derived stem cell, *ΔCSI* difference in cell signal intensity, *IQR* interquartile range, *MCAo* middle cerebral artery occlusion

### MRI analysis of infarct size 48 h post MCAo

**Fig. 2:**
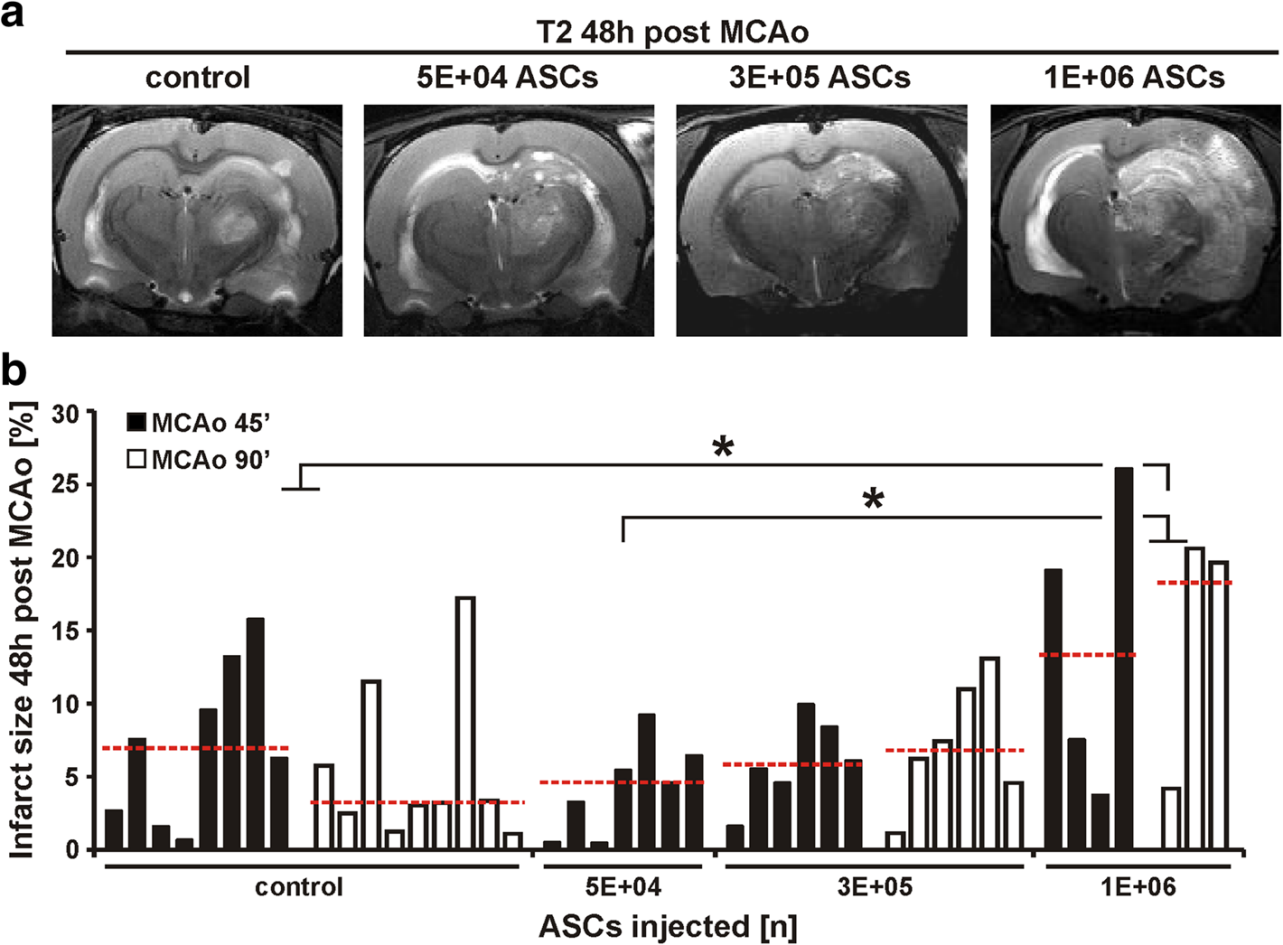
MRI analysis of infarct size 48 h post-MCAo. **a** Representative T2-weighted images displaying infarction 48 h post MCAo are the corresponding images to the T2*-weighted images shown in Fig. 1 and are derived from the same MRI slices and animals. **b** Differences in infarct volumes obtained 48 h post MCAo are shown for all treatment groups. Groups are the same as in Fig. 1. The median for each group is indicated as a *red dotted line*. Each column represents the infarct volume as a percentage of total individual brain volume for one animal. *p < 0.05 for 1 × 10^6^ ASC group vs. control group and 5 × 10^4^ ASC group. *ASC* adipose tissue - derived stem cell, *ΔCSI* difference in cell signal intensity, *MCAo* middle cerebral artery occlusion

To evaluate the effect of ASC engraftment on infarction, T2-weighted MR images were obtained from the same animals and MRI slices. Forty-eight hours after MCAo and ASC injection, the lesion area in the ipsilateral side of the brain was visible as a hyperintense signal and increased visibly after engraftment of 1 × 10^6^ cells (Fig. 2a).

To evaluate the effect of ASC engraftment on infarct size, the infarct volumes were determined as a percentage of the individual total brain volume (data range, medians, and IQRs for infarct size are shown in Table 1). The results revealed no influence of occlusion time on infarct volume in the control group and the group receiving 3 × 10^5^ cells. For animals receiving 1 × 10^6^ cells, the occlusion times could not be compared due to restricted data for the group undergoing MCAo for 90 min; however, due to comparable infarct volumes in the first-mentioned groups, a statistical comparison for every group was performed independently of occlusion time. Infarct sizes were significantly larger for animals receiving 1 × 10^6^ cells compared to the control group (*p* < 0.05) or animals receiving 5 × 10^4^ cells (*p* < 0.05). Infarct sizes in the group receiving 3 × 10^5^ ASCs lay between those of control animals or animals injected with 5 × 10^4^ cells and the group receiving 1 × 10^6^ cells (Fig. 2b).

### T2* re-analysis and cell signal development

**Fig. 3:**
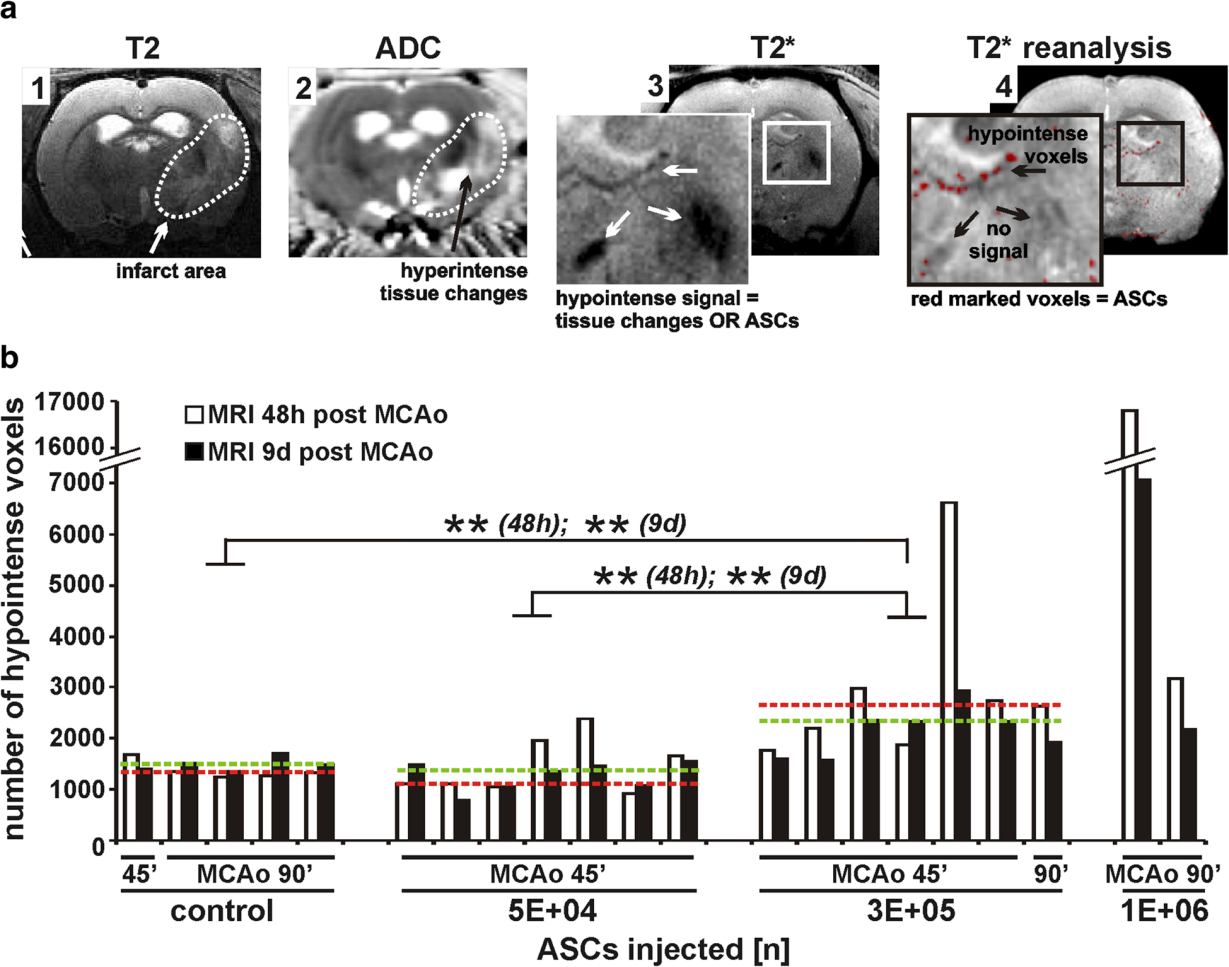
T2* re-analysis and cell signal development. **a** Signal pattern changes within 9 days post MCAo in the infarct area (*dotted line*, image 1) and consequential hyperintense signals displaying tissue changes as shown by the ADC map (image 2) are presented. Resulting ambiguous T2*-weighted hypointense signals (image 3) were re-analysed, leaving specific ASC-derived hypointense voxels (image 4). **b** Differences in voxel number obtained 48 h and 9 days post MCAo are shown for all treatment groups. Groups were controls (*n* = 5; *n* = 1 for MCAo = 45 min; *n* = 4 for MCAo = 90 min), and animals treated with 5 × 10^4^ ASCs (*n* = 7; all MCAo = 45 min), 3 × 10^5^ ASCs (*n* = 7; *n* = 6 for MCAo = 45 min; *n* = 1 for MCAo = 90 min), and 1 × 10^6^ ASCs (*n* = 2; all MCAo = 90 min). The median for each group is indicated as a *red dotted line* for voxel numbers obtained 48 h post MCAo and as a *green dotted line* for voxel numbers obtained 9 days post MCAo. Each pair of columns represents values of one animal and two MRI time points. **p < 0.01 for 3 × 10^5^ ASC group vs. control group and 5 × 10^4^ ASC group. *ADC* apparent diffusion coefficient, *ASC* adipose tissue - derived stem cell, *MCAo* middle cerebral artery occlusion

To investigate the time-course of ASC-derived signal intensity, an additional MRI analysis was carried out 9 days post MCAo in the subacute to chronic stage of infarct development. The results revealed signal pattern changes within the infarcted area (Fig. 3a; dotted line, image 1) visible as hyperintense signals and most likely deriving from development of vasogenic oedema, cell lysis, and tissue cavitation, respectively, as shown by the ADC map (Fig. 3a, image 2). This resulted in ambiguous T2*-weighted hypointense signals indicating iron-labelled ASCs or incipient tissue cavitation (Fig. 3a, image 3) which required a re-analysis, leaving specific ASC-derived hypointense voxels (Fig. 3a, image 4).

To evaluate possible changes in cell number between both MRI examinations, an assessment of T2*-derived voxel number was performed for both time points (data range, medians, and IQRs for voxel numbers are shown in Table 2). Due to the fact that not every animal underwent both MRI examinations and voxel analysis data are restricted to smaller populations compared to Fig. 1, groups could not be compared for MCAo duration but were distinguished in the figures. Animals receiving 3 × 10^5^ cells displayed significantly higher voxel numbers than control animals as well as animals receiving 5 × 10^4^ ASCs in both MRI examinations (*p* < 0.01). Voxel numbers did not change significantly between the two MRI scans except for one animal in the 3 × 10^5^ and one in the 1 × 10^6^ cell group. Because of insufficient data in the 1 × 10^6^ cell group, animals injected with 1 × 10^6^ cells could not be analysed for statistics (Fig. 3b).

**Table 2:** Group sizes, data range, median, and IQR for voxel numbers obtained 48 h and 9 days post-MCAo

Variable			Voxel numbers					
			48 h post MCAo			9 days post MCAo		
Treatment (number of ASCs)	MCAo (min)	Group size (*n*)	Data range	Median	IQR	Data range	Median	IQR
Control	45	1	1250 to 1686	1331	267	1361 to 1706	1490	230
	90	4						
5 × 10^4^	45	7	931 to 2380	1106	875	793 to 1559	1369	433
3 × 10^5^	45	6	1774 to 6611	2636	1132	1576 to 2943	2330	744
	90	1						
1 × 10^6^	90	2	16795 and 3168	–	–	7841 and 2162	–	–

*ASC* adipose tissue-derived stem cell, *IQR* interquartile range, *MCAo* middle cerebral artery occlusion

### Residual infarct size 9 days post MCAo and cell migration analysis

**Fig. 4:**
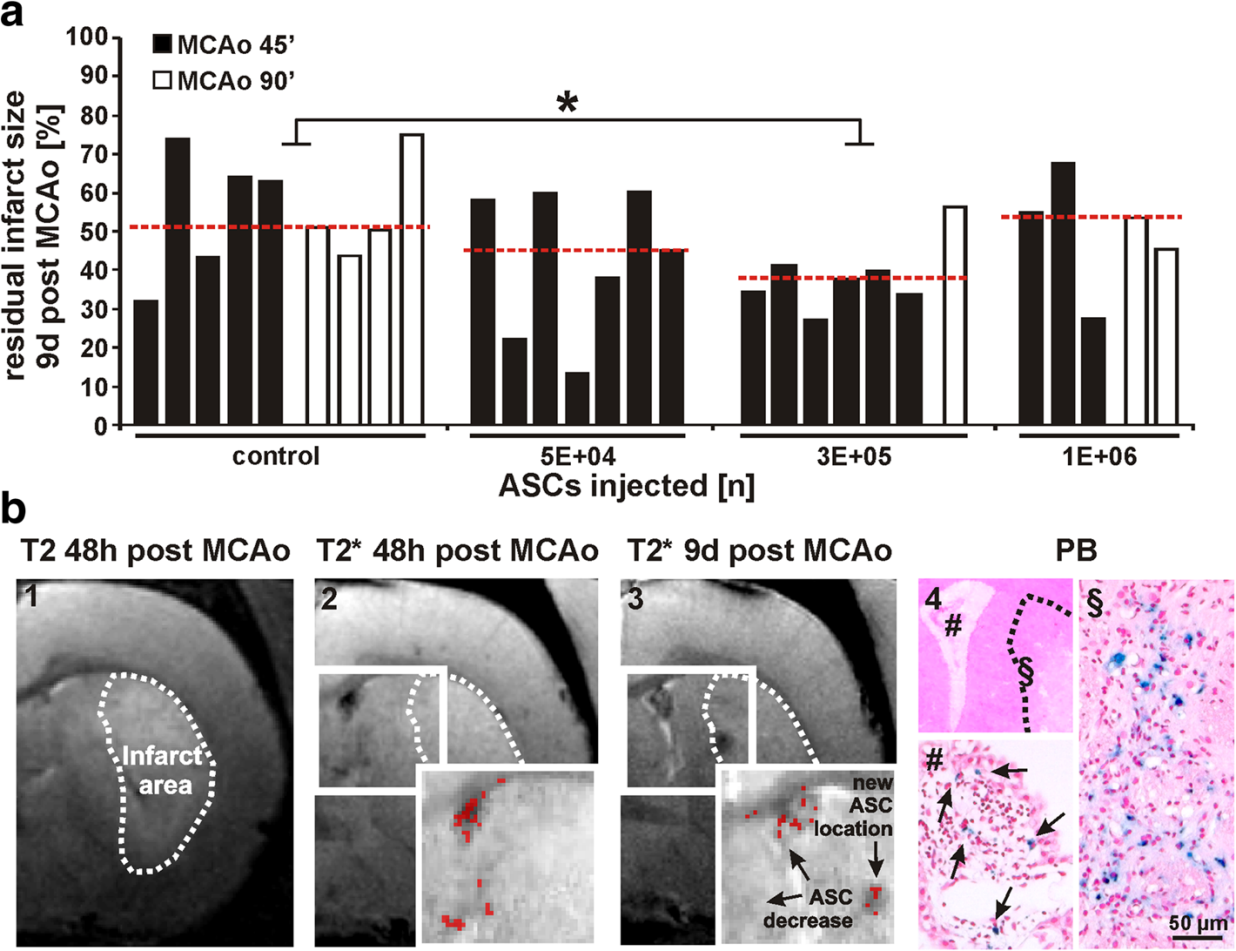
Residual infarct size 9 days post MCAo and cell migration analysis. **a** Differences in residual infarct volumes obtained 9 days post MCAo are shown for all treatment groups. Groups are controls (*n* = 9; *n* = 5 for MCAo = 45 min; *n* = 4 for MCAo = 90 min), and animals treated with 5 × 10^4^ ASCs (*n* = 7; all MCAo = 45 min), 3 × 10^5^ ASCs (*n* = 7; *n* = 6 for MCAo 45 min; *n* = 1 for MCAo = 90 min), and 1 × 10^6^ ASCs (*n* = 5; *n* = 3 for MCAo = 45 min; *n* = 2 for MCAo = 90 min). The median for each group is indicated as a *red dotted line*. Each column represents residual infarct size for one animal assessed as a percentage of the individual infarct size obtained 48 h post MCAo for each individual animal. *p < 0.05 for 3 × 10^5^ ASC group vs. control. **b** Migration activity in the ischaemic hemisphere (image 1, *dotted line*) of one animal treated with 3 × 10^5^ ASCs is shown. A strong hypointense signal in the right ventricle of the appending T2*-weighted image (image 2) obtained after 48 h decreases within 9 days post MCAo with a new hypointense spot appearing in the striatum close to the infarct area (image 3). Signals are confirmed by voxel analysis. Histological analysis of the same location reveals positive PB staining (blue signals, some supported by *arrows* in image 4, location # and §). *ASC* adipose tissue - derived stem cell, *MCAo* middle cerebral artery occlusion, *PB* Prussian blue

To investigate the long-term effect on infarct size, residual infarct volumes 9 days post stroke were determined as an individual percentage of the infarct size obtained 48 h post MCAo for each animal included in this analysis (data range, medians, and IQRs for infarct size are shown in Table 3). Due to the fact that not every animal underwent both MRI examinations the groups could not be compared for MCAo duration but were distinguished in the figures. Infarct volumes of the control group were comparable to those of the groups receiving 5 × 10^4^ or 1 × 10^6^ cells but differed significantly from the group receiving 3 × 10^5^ cells (*p* < 0.05), which showed visibly reduced infarct sizes (Fig. 4a).

**Table 3:** Group sizes, data range, median, and IQR for residual infarct sizes obtained 9 days post MCAo

Variable			Residual infarct size (%)		
Treatment (number of ASCs)	MCAo (min)	Animals (*n*)	Data range	Median	IQR
Control	45	5	32.16 to 74.84	51.1	25.53
	90	4			
5 × 10^4^	45	7	13.52 to 60.17	45.02	37.7
3 × 10^5^	45	6	27.16 to 56.4	37.84	7.14
	90	1			
1 × 10^6^	45	3	27.31 to 67.46	53.6	24.89
	90	2			

*ASC* adipose tissue-derived stem cell, *IQR* interquartile range, *MCAo* middle cerebral artery occlusion

Due to improved stroke outcome in animals receiving 3 × 10^5^ cells, the migration activity was assessed by comparing MRI signals visible in T2*-weighted images between both MRI examinations. Differences in hypointense signals indicated cell movement towards the ischaemic border zone as shown in one animal from the group receiving 3 × 10^5^ ASCs. A strong T2*-weighted hypointense signal in the right ventricle of the ischaemic hemisphere 48 h post MCAo (Fig. 4b, images 1 and 2), which we also verified by voxel reanalysis, underwent signal loss as shown by MRI analysis 9 days post stroke in the same animal and MRI slide. At the same time, a hypointense spot revealed as an ASC-derived signal appeared at the ischaemic border zone in the striatum (Fig. 4b, image 3). Under histology we identified the same location and examined it for possible stem cell signals using PB staining. The staining revealed positive signals for VSOP-derived iron oxide particles (Fig. 4b, image 4).

## Discussion

In this study, we investigated the influence of three different IA injected doses of ASCs on stroke outcome and stem cell homing. This was done via in vivo 9.4-T MRI in a rat model of MCAo-induced stroke.

Over the last few years, many stem cell studies focusing on stem cell administration, homing and tracking, have mainly reported on the methological details, e.g. the preparation of cell suspension of different stem cells, injection parameters, stem-cell labelling, and tracking or the time of stem-cell delivery [17, 28–33]. However, the question of the right volume of stem cells being used for transplantation was only addressed in one study injecting different numbers of human neural progenitor cells (NPCs) into the right striatum of male Wistar rats [34]. Darsalia and colleagues observed that transplantation of greater numbers of grafted NSCs did not result in a greater number of surviving cells or increased neuronal differentiation. However, since a different type of stem cell was used and the experimental settings differed, the results are only partially comparable to ours. To our knowledge, we are the first study raising the question of whether, and to what extent, the number of IA engrafted stem cells influences the parameters mentioned above and if there is an optimal number recommended for this administration.

Although the central nervous system (CNS) is an immune-privileged region, a xenogenic transplantation of human ASCs in a rat model may induce reactions of the rodent immune system. This is a problem affecting all studies testing human stem cells in rodent systems; however, even allogeneic transplantation is being discussed as including the risk of immune rejection [35]. There are conflicting reports regarding the need for immunosuppressive therapy after xenograft stem-cell therapy for cell survival and differentiation, and it is still unclear whether ASCs, despite their immunomodulatory capacities, may elicit systemic immune reactions [36]. Similar to our previous work, where we were able to observe therapeutic efficacy and stimulation of endogenous repair, we have stuck to this protocol and avoided immunosuppressive therapy, and thus possible potential effects on stroke progression and cell distribution [37]. Furthermore, our investigations where designed as a proof-of-concept study with a survival time of only 14 days, which is short compared to the 6 weeks used by, for example, Hovakimyan et al. [36].

Studies show that the injured brain is more favourable for stem cell migration than is the intact tissue. Evidence from transplantation studies indicate that the intact adult brain has a limited capacity to direct the differentiation of transplanted stem cells, whereas the developing brain and injured brain express morphogenic cues that appear to foster donor plasticity [38]. Studies transplanting NPCs either directly or IV have demonstrated that cells need a triggering signal to undergo targeted migration in the rodent CNS and that neural NPCs transplanted into the un-injured adult rodent brain undergo only limited and non-targeted migration [14, 39]. Another study using two sources of murine bone marrow stromal cells (BMSCs) demonstrated a seven-to nine-fold increase in the number of adherent BMSCs that accumulate in cerebral venules after focal transient ischaemia compared to sham-operated mice [40]. However, the aim of the study was to investigate the effect of different stem cell numbers on engraftment rate and stroke outcome after transient brain ischaemia. Therefore, we only included animals with MCAo-induced stroke.

In the past few years the question of how the biodistribution of systemically applied MSCs is regulated has also included discussion about the advantages and disadvantages of IV and IA administration. A very important question in this therapeutic field is how to bring enough stem cells to the targeted tissue. IV injection is a minimally invasive, and therefore clinically highly relevant, technique. On the other hand, it bears the disadvantage of delayed and poor engraftment due to long-distance migration and filtering organs; it was shown by several studies that up to 80% of injected cells distribute to a variety of non-targeted tissues, with most cells being trapped in the lungs, followed by the liver and spleen [41–44]. Consistently, studies comparing IV and IA administration of NPCs and BMSCs observed no, or a poor and late, engraftment in the ischaemic brain after IV injection [14, 16]. A second factor to bear in mind is the formation of emboli in lung vessels [42]. Cell culture-expanded MSCs are relatively large cells with an average size of 30 μm. Therefore, obstructive events during lung passage are expected after MSC administration which is supported by their relatively high adhesion capacity and their tendency to aggregate, leading to clogging of capillaries [42, 45, 46]. Contrary to the observation of MSC retention in the lungs, one study analysing the biodistribution of BMSCs after IA injection found a decreased deposition in the lungs and increased uptake in other organs, especially in the liver [47]. With regard to engraftment time and rates, studies comparing both application routes have demonstrated a major association between IA application and the early and increased accumulation in therapeutic target tissues [14, 16]. However, this was associated with an impeded cerebral blood flow and higher mortality rates. In conclusion, the IA route of administration seems to be effective in avoiding pulmonary entrapment and may thus improve the biodistribution and bioavailability of transplanted MSCs in clinically relevant issues, e.g. for tissue repair.

As mentioned, IA delivery was reported to result in higher mortality rates compared to the IV application [14, 16–18]. Since 1 × 10^6^ stem cells are the standard amount for transplantation in rodents, we also included this number in our study, but we had tremendous difficulties obtaining enough data due to mortality rates being twice as high as for the other groups. Since mortality rates were only 0–33% in the other groups we cannot confirm the idea of IA delivery increasing the mortality rate per se. We hypothesize the outcome may depend on the cell amount because, in the case of IA injection, a larger cell number could be brought to the brain more quickly, bypassing systemic organs. As a consequence this may lead to increased mortality due to impeded CBF [16]. But since we have not examined other organs where cells are typically found after systemic application (e.g. lungs) we cannot exclude the possibility of pulmonary embolism resulting from cells bypassing the brain.

The comparison of infarct volumes reflected the results we obtained from mortality rates since application of 1 × 10^6^ ASCs led to an increase in infarct size. This corresponds to Lu et al. who obtained similar results in two dogs after injection of 3 × 10^6^ cells [15]. In addition to the impeded blood flow observed by Walczak et al. [16], cell injection could lead to occlusion of cerebral microvessels and thus to comprised microvascular circulation and increased infarct size. Another possibility leading to disturbed blood flow is the fact that MSCs have been shown to possess blood clotting properties by triggering the pro-coagulatory cascade via surface expression of tissue factors. This in turn may cause thromboembolism promoting mechanical vascular obstruction [48–51]. This closes the loop to immunological complications of intravascular stem-cell applications derived by strong immunosuppressive properties that may amplify the post-stroke immune deficiency syndrome and hence the risk for infections [52–55]. However, the majority of pre- and clinical data support the notion that embolic events are very rare and in the majority of cases are a mild side effect of intravenous infusion, which can be easily circumvented by adding anticoagulants such as heparin during cell infusion [56].

In our study, an occlusion time-dependent effect could not be observed possibly due to the model-adherent variability in infarct sizes we obtained. At 9 days post MCAo, the mean infarct size in the 3 × 10^5^ ASC group decreased about 30% whereas infarct sizes in the other groups were comparable to the infarct size in the control group. Where animals received 1 × 10^6^ ASCs this is consequential due to higher initial infarct sizes, but for the group receiving 5 × 10^4^ ASCs one could speculate that this cell amount is not high enough to affect stroke recovery positively. A positive reduction of infarct size up to 54% could also be observed in previous studies after IA injection in Wistar and Sprague-Dawley rats, but since 1 × 10^6^ cells or more were used this is contrary to what we found [18, 57]. However, mortality rates were not discussed and experimental settings were slightly different which, in general, makes it difficult to compare results.

As described previously and confirmed in vivo, T2*-weighted images revealed engrafted cells scattering discretely throughout the entire lesion area [14–16]. This agrees with the theory that transplanted cells are attracted by, and interact with, tissue regions undergoing degeneration and reorganization [58]. In the acute stage, 48 h post stroke differences in CSI depended on the injected cell number which we could also sensitively observe by 9.4-T MRI. However, cell signal analysis in the subacute to chronic stage 9 days post MCAo was difficult due to tissue changes initiated by stroke and followed by vasogenic oedema, cell lysis, and the beginning of tissue cavitation, as indicated by the ADC maps [59–61]. Therefore, T2*-weighted signals had to be re-analysed and ASC-derived signals be identified. We could solve this problem by performing a re-analysis of T2*-weighted images for reliable identification of voxels deriving from ASC signals. After successful voxel analysis we observed no difference in voxel numbers between both MRI examinations in most of the animals that we analysed. Contrary to our findings, Lu et al. observed a clear signal attenuation 14 days post MCAo using 3-T MRI after transplantation of VSOP-labelled MSCs [15]. Since the MRI detection threshold of labelled cells depends on particle concentration per cell, cell density after integration, and MRI parameters such as field strength, 3-T MRI analysis might involve detection limits due to lower contrast-to-noise ratio compared to 9.4-T imaging [62, 63]. As Lu et al. mentioned, this may lead to the assumption of signal loss.

Since we could not observe a signal loss 9 days post MCAo that would have implied cell degradation, we assumed that this result may indicate predominantly vital ASCs, with them being potentially able to proliferate and migrate actively within the brain parenchyma. Subsequent histological analysis using PB staining as a reliable tool for VSOP-labelled cell visualization revealed PB-positive locations being also positive for VSOP fluorescence and human markers. The fact that almost all the cell cytoplasm of VSOP-labelled ASCs were distinctively blue showed that neither excessive cell degradation nor excessive proliferation activity had taken place because this would have led to weakened staining results in daughter cells as shown in our obtained growth curves. We further tested proliferation activity using Ki67. Positive signals indicated that at least some ASCs had started proliferation. However, our findings do not exclude the possibility of cell degradation taking place or the presence of resting cells at other locations. Despite some challenges due to tissue changes, we consider T2*-imaging as a very promising tool that might also offer further valuable information about tissue oxygenation in animal and human studies for future applications [64, 65].

Because of the observed reduction in infarct size in the group receiving 3 × 10^5^ ASCs, we were interested in migration activity within the brain parenchyma leading to promotion of brain remodelling and neurological recovery [12]. From early observations in the literature we know that stem cells are able to migrate to an ischaemic area very soon after transplantation [66–68]. After analysis of the MRI data of all our groups we found a strong hypointense signal in the right ventricle and striatum of one animal treated with 3 × 10^5^ cells. This signal was confirmed by voxel analysis and was attenuated between 48 h and 9 days, while a new signal appeared right at the ischaemic border. Histological analysis of this animal revealed residual ASCs in the ventricle and “new” ASCs at the ischaemic border at this location. Although this observation looks very promising, especially due to a decline in infarct volume in the 3 × 10^5^ cell group, the limitation must be noted that this observation could only be made in one animal. The reason for this can probably be found in the fact that locally and temporarily highly limited processes such as cell migration are difficult to capture and that more consecutive MRI examinations and more animals per group may be necessary to solidify our observation.

We are aware of limitations in our study; this applies in particular to the group sizes. As we have already mentioned, the 1 × 10^6^ cell group showed mortality rates twice as high as the other groups. Since mortality rates are rarely discussed in comparable settings, this was surprising for us. The only indications in the literature can be found in a study of Li et al. who observed a mortality rate of 41% in Wistar rats being injected IA with 1 × 10^6^ NPCs 24 h after transient 2 h ischaemia, as well as by Walczak et al. who observed a mortality rate of 67% in Wistar rats being injected IA with 1 × 10^6^ BMSCs 30 min after transient 2 h ischaemia [14, 16]. Furthermore, we had limited numbers of animals being examined for voxel analysis and residual infarct size post stroke since not every animal underwent both MRI examinations. Nevertheless, the essential statements concerning the effect of different stem cell numbers on engraftment rate and stroke outcome after transient brain ischaemia could be validated with the numbers we had. For upcoming studies we will have to take the limitations, e.g. mortality rate, of this study into account.

Future histological analysis of the animals will pursue interesting issues involving the question of cell location and distribution within the brain after cell delivery and how the initially injected cell number affects the amount and percentage of cells migrating into the brain.

## Conclusions

In conclusion, although IA delivery of SCs is a more invasive method compared to IV injection, it offers the advantage of direct targeting of the damaged tissue. Since cells are solid, they likely have the big disadvantage of provoking impeded CBF and vessel occlusion after injection of high cell numbers. In therapeutic doses, however, systemically delivered SCs may promote brain remodelling and induce functional neurological recovery. Therefore, the future difficulty will be to achieve high cellular engraftment in patients receiving stem cell treatment without the side effects mentioned above. The correct amount of stem cells could be a first step towards the use of an efficient chemoattractive gradient released at the site of the brain lesion.

## Additional files

Additional file 1: Supplemental material. (DOCX 18 kb)

Additional file 2: Figure S1. Influence of VSOP labelling on cell vitality and growth behaviour. LDH release did not differ for unlabelled (negative control) and VSOP-labelled ASCs. Both differed significantly from positive control. *p < 0.05 for negative control and VSOP-labelled cells vs. positive control (A). Growth curves and fraction of dead cells did not differ between labelled and unlabelled cells at any time (diagram). Growth curves of vital cells increased significantly during the observation period. VSOP density in cytoplasm decreased within 9 days (images). *p < 0.05 for increased cell growth up to 14 days for VSOP-labelled and unlabelled ASCs (B). *ASC* adipose tissue-derived stem cell, *LDH* lactate dehydrogenase, *VSOP* very small superparamagnetic iron oxide particle﻿s. (TIF 6166 kb)

Additional file 3: Figure S2. Identification and localisation of VSOP-labelled ASCs via PB staining and VSOP fluorescence. Representative images of a PB-stained brain section 14 days post-MCAo of an animal treated with ASCs and enlarged locations within the brain slice. A single ASC is visualized (image 3) and distinguished from the unlabelled rat brain cell. ASCs correlate with locations of VSOP fluorescence obtained in a neighbour slice (image 8a, blue = DAPI stained cell nuclei, green = VSOPs). *ASC* adipose tissue-derived stem cell, *DAPI* 4',6-﻿d﻿iamidino-2-phenylindole, *MCAo* middle cerebral artery occlusion, *PB* Prussian blue, *VSOP* very small paramagnetic iron oxide particles. (TIF 7415 kb)

Additional file 4: Figure S3. Identification and viability testing of ASCs via staining of human mitochondria and Ki67. Representative images of PB-positive areas (left images) co-localized with locations positive for human mitochondria (B) and Ki67 (C) (DAB-positive signal, middle images) in neighbour slices whereas negative controls showed no DAB signal (right images). *ASC* adipose tissue-derived stem cell, *DAB* 3,3'-diaminobenzidine, *PB* Prussian blue. (TIF 7971 kb)

## Abbreviations

ADC: Apparent diffusion coefficient
ASC: Adipose tissue-derived stem cell
BMSC: Bone marrow stromal cell
CBF: Cerebral blood flow
CNS: Central nervous system
CSI: Cell signal intensity
DAB: Diaminobenzidine
DAPI: 4′,6-Diamidino-2-phenylindole
DWI: Diffusion-weighted image
ECA: External carotid artery
EDTA: Ethylenediaminetetraacetic acid
FLIRT: FMRIB’s Linear Image Registration Tool
FSL: FMRIB’s Software Library
IA: Intra-arterially
IQR: Interquartile range
IV: Intravenously
LDH: Lactate dehydrogenase
MCAo: Middle cerebral artery occlusion
MRI: Magnetic resonance imaging
MSC: Mesenchymal stem cell
NPC: Neural progenitor cell
PB: Prussian blue
PBS: Phosphate-buffered saline
ROI: Region of interest
SC: Stem cell
SD: Standard deviation
SS-EPI: Single-shot spinecho echo-planar imaging
TRIS: Tris(hydroxymethyl)aminomethane
VSOP: Very small superparamagnetic iron oxide particles
ΔCSI: Difference in cell signal intensity
