# Supplementary material for: The effect of adipose tissue-derived stem cells in a middle cerebral artery occlusion stroke model depends on their engraftment rate
Source: Stem Cell Res Ther. 2017 Apr 26;8:96. doi: 10.1186/s13287-017-0545-y (PMC5407025; doi:10.1186/s13287-017-0545-y)
Supplement: Supplementary file 1 — Supplemental material. (DOCX 18 kb) [file 13287_2017_545_MOESM1_ESM.docx]

**Supplemental material**

**Results**

1. Influence of VSOP labelling on cell growth and vitality *in vitro*

LDH release of negative control ranged from 0.24 to 0.77 (median = 0.41; IQR = 0.39) and was similar to VSOP labelled ASCs where LDH release ranged from 0.45 to 0.61 (median = 0.51; IQR = 0.11). Both differed significantly from positive control ranging from 0.88 to 1.32 (median = 1.26; IQR = 0.27) (p < 0.05) (Fig. S-1A). Growth curves and fraction of dead cells did not differ between labelled and unlabelled ASCs at any time. Mean ASC number for controls increased from 19750+/-6418 to 558157+/-144725 cells. The fraction of dead ASCs ranged from 8750+/-7041 to 80510+/-53829 cells. Cell number for VSOP labelled ASCs increased from 24754+/-8919 to 728624+/-156603 cells. The fraction of dead ASCs ranged from 6996+/-4742 to 106168+/-128725 cells. Growth curves of vital ASCs increased significantly during observation period (p < 0.05). VSOP density in cell cytoplasm decreased within 9 days (Fig. S-1B).

1. Mortality rates

71.4% (10 of 14; MCAo = 45 min) and 75% (9 of 12; MCAo = 90 min) of animals in the group receiving 1E+06 ASCs died within 24h post-surgery. In the 3E+05 cell group mortality rates were 33.3% (3 of 9 for each MCAO 45 and 90 min). No animal receiving 5E+04 cells (all MCAO 45 min) died. In the control groups no animal with occlusion time of 45 min died whereas it was 30.8% (4 of 13) when MCAo was 90 min.

1. Identification, localisation and viability testing of ASCs

After PB staining single ASCs could be visualized sensitively and distinguished from unlabelled brain cells as shown by PB positive cytoplasm (image 3). ASCs were located close to the lesion area in the ipsilateral side of the brain and correlated with locations of VSOP fluorescence obtained in the neighbour slice (image 8a) (Fig. S-2).

Staining of human mitochondria confirmed our observations and verified PB positive areas as cells of human origin (Fig. S-3A). Positive signal observed after staining of Ki67 as proliferation marker strongly indicates vital proliferating ASCs (Fig. S-3B).
