# Supplementary figures and images for: The effect of adipose tissue-derived stem cells in a middle cerebral artery occlusion stroke model depends on their engraftment rate
Source: Stem Cell Res Ther. 2017 Apr 26;8:96. doi: 10.1186/s13287-017-0545-y (PMC5407025; doi:10.1186/s13287-017-0545-y)

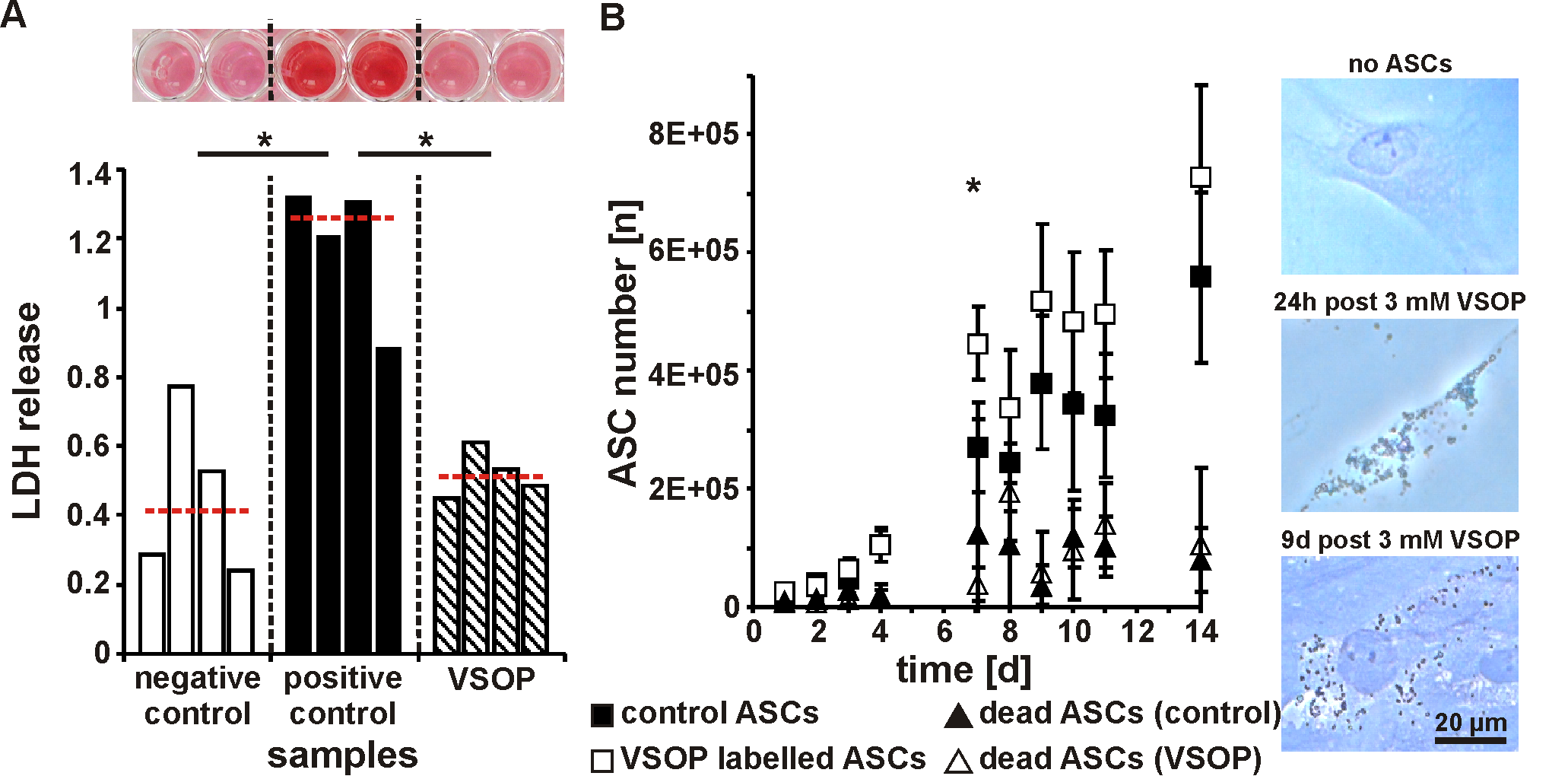

Supplement: Supplementary file 2 — Influence of VSOP labelling on cell vitality and growth behaviour. LDH release did not differ for unlabelled (negative control) and VSOP-labelled ASCs. Both differed significantly from positive control. *p < 0.05 for negative control and VSOP-labelled cells vs. positive control (A). Growth curves and fraction of dead cells did not differ between labelled and unlabelled cells at any time (diagram). Growth curves of vital cells increased significantly during the observation period. VSOP density in cytoplasm decreased within 9 days (images). *p < 0.05 for increased cell growth up to 14 days for VSOP-labelled and unlabelled ASCs (B). ASC adipose tissue-derived stem cell, LDH lactate dehydrogenase, VSOP very small superparamagnetic iron oxide particle﻿s. (TIF 6166 kb) [file 13287_2017_545_MOESM2_ESM.tif]

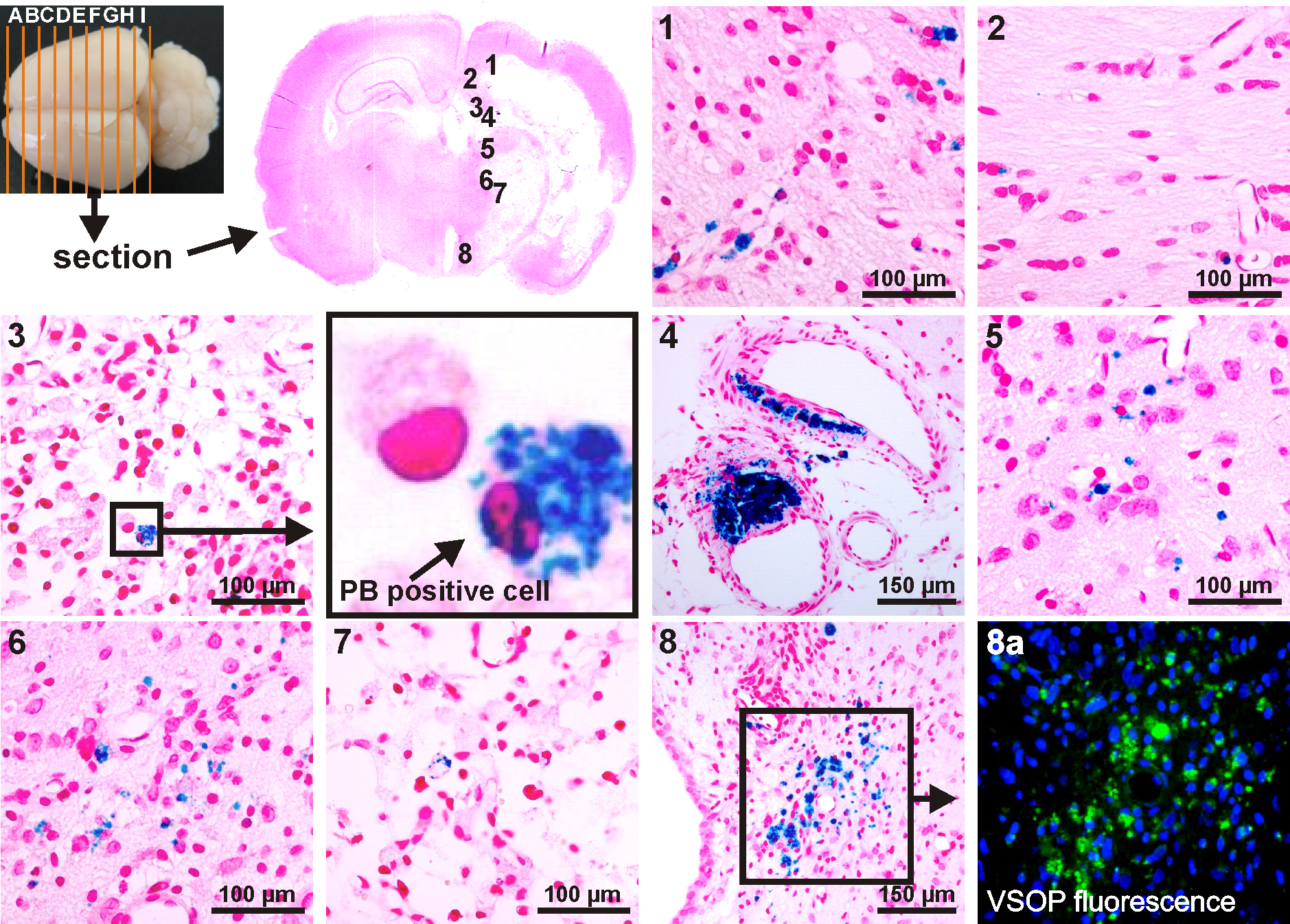

Supplement: Supplementary file 3 — Identification and localisation of VSOP-labelled ASCs via PB staining and VSOP fluorescence. Representative images of a PB-stained brain section 14 days post-MCAo of an animal treated with ASCs and enlarged locations within the brain slice. A single ASC is visualized (image 3) and distinguished from the unlabelled rat brain cell. ASCs correlate with locations of VSOP fluorescence obtained in a neighbour slice (image 8a, blue = DAPI stained cell nuclei, green = VSOPs). ASC adipose tissue-derived stem cell, DAPI 4',6-﻿d﻿iamidino-2-phenylindole, MCAo middle cerebral artery occlusion, PB Prussian blue, VSOP very small paramagnetic iron oxide particles. (TIF 7415 kb) [file 13287_2017_545_MOESM3_ESM.tif]

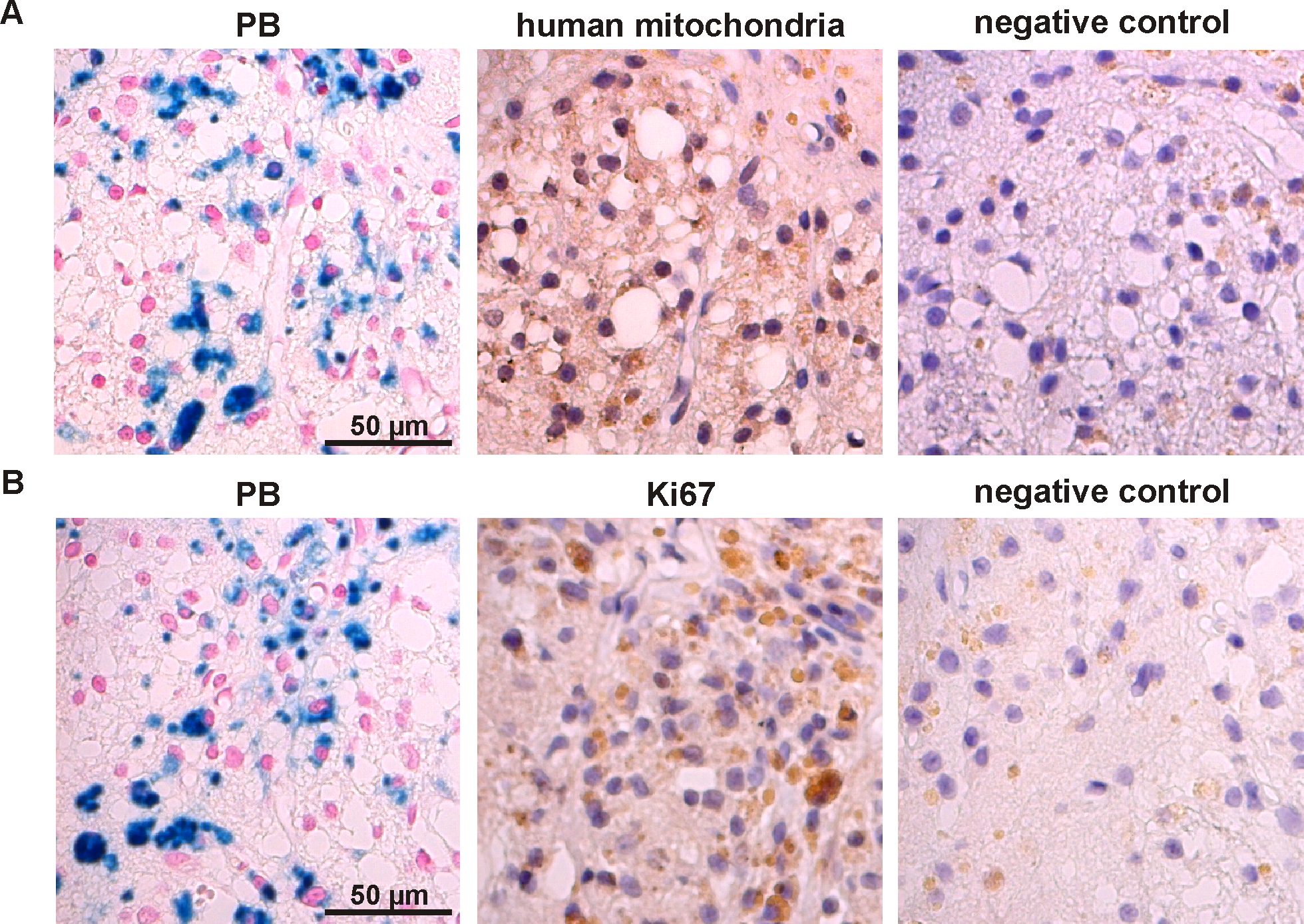

Supplement: Supplementary file 4 — Identification and viability testing of ASCs via staining of human mitochondria and Ki67. Representative images of PB-positive areas (left images) co-localized with locations positive for human mitochondria (B) and Ki67 (C) (DAB-positive signal, middle images) in neighbour slices whereas negative controls showed no DAB signal (right images). ASC adipose tissue-derived stem cell, DAB 3,3'-diaminobenzidine, PB Prussian blue. (TIF 7971 kb) [file 13287_2017_545_MOESM4_ESM.tif]
